# Pregnancy-Related Information Seeking and Sharing in the Social Media Era Among Expectant Mothers: Qualitative Study

**DOI:** 10.2196/13694

**Published:** 2019-12-04

**Authors:** Chengyan Zhu, Runxi Zeng, Wei Zhang, Richard Evans, Rongrong He

**Affiliations:** 1 College of Public Administration Huazhong University of Science and Technology Wuhan China; 2 Center for Communication and Social Development School of Journalism and Communication Chongqing University Chongqing China; 3 Smart Health Institute School of Medicine and Health Management, Tongji Medical College Huazhong University of Science and Technology Wuhan China; 4 College of Engineering Design and Physical Sciences Brunel University London London United Kingdom; 5 School of Public Administration Sichuan University Chengdu China

**Keywords:** pregnant women, social media, information seeking, consumer health information, China

## Abstract

**Background:**

Social media has become the most popular communication tool used by Chinese citizens, including expectant mothers. An increasing number of women have adopted various forms of social media channels, such as interactive websites, instant messaging, and mobile apps, to solve problems and obtain answers to queries during pregnancy. Although the use of the internet by pregnant women has been studied extensively worldwide, limited research exists that explores the changing social media usage habits in China, where the 1 child policy ended in 2015.

**Objective:**

This study aimed to (1) present the status quo of pregnancy-related information seeking and sharing via social media among Chinese expectant mothers, (2) reveal the impact of social media usage, and (3) shed light on pregnancy-related health services delivered via social media channels.

**Methods:**

A qualitative approach was employed to examine social media usage and its consequences on pregnant women. A total of 20 women who had conceived and were at various stages of pregnancy were interviewed from July 20 to August 10, 2017. Thematic analysis was conducted on the collected data to identify patterns in usage.

**Results:**

Overall, 80% (16/20) of participants were aged in their 20s (mean 28.5 years [SD 4.3]). All had used social media for pregnancy-related purposes. For the seeking behavior, 18 codes were merged into 4 themes, namely, gravida, fetus, delivery, and the postpartum period; whereas for sharing behaviors, 10 codes were merged into 4 themes, namely, gravida, fetus, delivery, and caretaker. Lurking, small group sharing, bad news avoidance, and cross-checking were identified as the preferred patterns for using social media. Overall, 95% (19/20) of participants reported a positive mental impact from using social media during their pregnancy.

**Conclusions:**

It is indisputable that social media has played an increasingly important role in supporting expectant mothers in China. The specific seeking and sharing patterns identified in this study indicate that the general quality of pregnancy-related information on social media, as well as Chinese culture toward pregnancy, is improving. The new themes that merge in pregnancy-related social media use represent a shift toward safe pregnancy and the promotion of a more enjoyable pregnancy. Future prenatal care should provide further information on services related to being comfortable during pregnancy and reducing the inequality of social media–based services caused by the digital divide.

## Introduction

### Social Media Engagement in Pregnancy-Related Topics Worldwide

Pregnancy-related information is in great demand worldwide. A qualitative study conducted in the United States implied that pregnancy-related information, such as that relating to healthier lifestyles, childbirth, infant care, and being a good mother, were major unmet needs of pregnant women, where technologies for use during pregnancy could be significantly improved [1]. Similarly, China has faced such challenges because of its comparatively lower health literacy rates [2]. This situation has intensified since the termination of the 1 child policy in 2015, with the population of pregnant women being on the rise as well as their information needs; this is especially true in the case of older pregnant women.

The internet has been widely used as an effective tool to meet health information needs across the world, and pregnancy-related information is no exception [3,4]. A systematic review on the internet usage by pregnant women found that fetal development and nutrition during pregnancy were highly mentioned topics [3]. In China, pregnant women mainly evaluate the information obtained via the internet by consulting various online sources before drawing conclusions, and they perceive information found on the internet to be generally reliable [5]. With the evolution of internet-based technologies, social media has penetrated all walks of human life as well as prenatal care [6]. Social media is defined as “a group of Internet-based applications that build on the ideology and technological foundations of Web 2.0” [7,8], which enables the creation and exchange of information, allowing users to become the content generators rather than solely the receivers of content. Social media forms are not limited to websites but include blogs, online forums, online discussion boards, and various other apps or platforms. As an alternative source of pregnancy-related information, social media has become hugely popular as an instrument for obtaining desired health information, sharing concerns felt during pregnancy, and communicating with other pregnant women [9,10]. Compared with offline methods of communication, pregnancy-related information retrieved via social media is deemed much more convenient, detailed, practical, customized, and unbiased [9,11]. A survey conducted in Australia showed that almost three-quarters (73%) of expectant mothers had used pregnancy-related apps [12]. Similarly, the United States and European countries have evidenced the popularity of pregnancy-based social media usage [13,14]. In addition, several social media channels have featured *father-to-be* campaigns to encourage participation by expectant fathers during pregnancy [15].

### Pregnancy-Related Engagement in China’s Social Media Platforms

In China, research into online pregnancy-related information retrieval is limited, with currently available research mainly focused on statistics obtained from websites and descriptions for specific geographic populations. Gao et al [5] explored pregnancy-related information searches in southeastern China and found that more highly educated women tended to assign higher credibility to online health information. In addition, they also noticed that a relatively low percentage of participants would discuss the online health information they had found with their caregiver or health professionals, in-person. A recent systematic review of available literature found a low presence of strong evidence for low- and middle-income countries in terms of the impact resulting from internet usage by pregnant women, considering their large volume of users [16,17]. In China, the most frequently used pregnancy-related apps are *MeetYou* and *BabyTree*. Various types of pregnancy-related information across different stages of child birth have been integrated into 1 software application, as along with the ability to search for health information and engage in interactive discussion forums and shopping.

It is recognized that pregnant women have individual health information needs and preferred sources for identifying this information. By accessing health information on social media, pregnant women become better informed about their health condition. An increasing number of studies have started to reveal the prevalence of social media usage by pregnant women, including their adoption, usage patterns, and impact. However, little analysis has been conducted on the current status quo of seeking and sharing pregnancy-related information on social media, in the context of China. Furthermore, most recent studies have focused on developed countries when exploring the functionality and rationality for social media usage by pregnant women. To fill this gap, this research extensively examined the search strategies, sharing patterns, and their impact on expectant mothers in China. As searching for suitable pregnancy-related information or sharing relevant pregnancy-related information is deemed important for expectant mothers, this study provided insight into how pregnant women in China use social media and its corresponding effects. By using the results of this study, enterprises with a special interest in pregnant women can better tailor their online services. The findings also encourage public or authoritative medical institutes to offer more personalized pregnancy-related information services via social media and other communicative tools.

## Methods

To achieve the aims of this study, a qualitative methodology was adopted. Qualitative methods were chosen for their ability to obtain firsthand data that could shed light on the culture and context of online pregnancy-related information searches from the study population [18]. The consolidated criteria for reporting qualitative research checklist was adhered to when considering the most appropriate method of study [19].

### Study Design

Interviews were targeted toward expectant mothers in China who use social media to obtain and synthesize information during pregnancy; this included exploring information seeking and sharing behaviors and their consequences. The inclusion criteria for interviewees were as follows: aged over 18 years, married, located in China, have known about their pregnancy for a period of at least one month, and used social media for pregnancy-related purposes at least once in the past month. The exclusion criteria were as follows: not residing in China, aged under 18 years, unmarried, have known about their pregnancy for a period of less than one month, and did not use social media for pregnancy-related purposes at all during the past month. Participants were selected based on a snowball sampling process. Initially, potential participants were solicited from the pools of acquaintances of the researchers via social media posts (eg, QQ and WeChat), whereas new participants joined if existing participants nominated participants that met the inclusion criteria. This study followed common practices in China for protecting the privacy of participants. As a recent quantitative study [20] revealed that young, first time expectant mothers, with a higher education level (ie, bachelor’s degree or above) were more likely to use social media–based pregnancy apps, we prioritized participants who were of a young age, had a higher education degree, and were experiencing pregnancy for the first time.

The interviews were completed by the fifth author (HRR), who is a young lecturer in a university and mother of a 3-year-old child. She has used social media for more than 10 years, with extensive experience of seeking and sharing pregnancy-related information on social media across different pregnancy cycles. Before participation, all participants were informed of the study’s aim, process, and the benefits and potential risks of partaking in the research. All participants agreed to participate in the interview process and consented to the interview data being recorded anonymously. All interviews were conducted face-to-face at a comfortable place for the participants, mostly near or at their homes. The interview phase was conducted from July 20 to August 10, 2017. Each interview lasted for at least 30 min.

### Data Collection and Analysis

All interviews were conducted in Chinese. Interviews were recorded and were transcribed later for analysis. The interview framework included the following areas of questioning: (1) participants’ description of their background and frequency of using social media during pregnancy, (2) description of their social media use, particularly on pregnancy-related information seeking and sharing, and (3) the reported impact of social media usage during pregnancy. Questioning began by asking the participants’ demographic information, such as age, educational level, occupation, pregnancy cycle, and their daily use of social media during their pregnancy. The focus then shifted to their experience of pregnancy-related information seeking and sharing on social media channels. In this section, our study was interested in the types of pregnancy-related information that were sought or shared, including their preferred social media channels and with whom. The participants were also asked to recall a situation when they sought or shared their pregnancy-related information on social media. In the last section, participants were queried about their personal reflection on the social media use for pregnancy-related purposes. This focused on their physical health and mental health as well as their relationship with caretakers. Before each interview, a list of pseudonyms was provided to the participants to select 1 and use them for documentation to protect their identity, and the researchers made all efforts to secure the participants’ privacy throughout the entire study. Table 1 shows the characteristics of the participants.

All participants were in their first marriage. Furthermore, 80% (16/20) were aged in their 20s (mean 28.5 years [SD 4.3]), with 1 participant aged 20 years and 1 participant over 40 years. In this study, most participants were primigravida (pregnant for the first time; 18/20, 90%). In terms of the stage of pregnancy cycle, many (16/20, 80%) were in their last trimester (over 28 weeks), whereas only 1 woman was in the early stage of pregnancy. With regard to their educational background, 60% (12/20) had obtained at least a bachelor’s degree, whereas 20% (4/20) had achieved a high school–level education or equivalent. Overall, 65% (13/20) of participants were employed, with their occupation varying from primary, middle, high, or vocational school teacher; university lecturer; support staff; assistant researcher; bank associate; accountant; to traffic police officer. In addition, with regard to monthly family income, 50% (10/20) of participants earned over 10,000 Renminbi (RMB), whereas 5 (25%) participants earned less than 10,000 RMB.

For the analysis of the seeking and sharing behaviors, thematic analysis of the transcript was conducted using recommended procedures [21], which included (1) familiarization with the data, (2) generation of initial codes, (3) searching for codes, (4) reviewing themes, (5) defining and naming themes, and (6) writing the results. An initial coding framework was developed with randomly selected texts from 5 interviews and the codebook was updated after each analysis. The codes were checked rigorously to ensure their fit to the original text. The codes with shared themes were merged and modified, if required.

After the analysis of the 19th interview transcript, new data did not emerge from the data collection. To ensure confidence and verification of the data saturation, the researchers followed a recent practice [22] and conducted another interview for confirmation. For the 20th interview, no additional codes were generated. It was clear that the data provided a clear saturation point and the interviews were then ceased. In total, 21 codes and 5 themes were identified after a redundancy check and discussion.

**Table 1:** Characteristics of the participants (N=20).

Characteristics		Participants, n (%)
**Age (years)**		
	18-20	1 (5)
	21-30	16 (80)
	31-40	2 (10)
	>40	1 (5)
**Education level**		
	Bachelor’s degree or above	12 (60)
	College degree	3 (15)
	High school or equivalent	4 (20)
	Unrevealed	1 (5)
**Occupation**		
	Employed^a^	13 (65)
	Unemployed/housewife	7 (35)
**Monthly family income (Renminbi)**		
	≤10,000	5 (25)
	10,001-15,000	6 (30)
	15,001-20,000	4 (20)
	Unrevealed	5 (25)
**Childbearing history/parity**		
	Primipara	18 (90)
	Multipara	2 (10)
**Pregnancy cycle**		
	First trimester (≤12 weeks)	1 (5)
	Second trimester (13-27 weeks)	3 (15)
	Last trimester (>27 weeks)	16 (80)

^a^Their occupations were teachers (n=8), university support staff (n=1), research assistant (n=1), bank associate (n=1), accountant (n=1), and traffic police (n=1).

## Results

### General Overview

All participants reported using social media for pregnancy-related purposes, but with different intensities. When asked about their frequency of use in the past month, 80% (17/20) reported that they used social media almost every day and the others reported less usage frequency, ranging from every other day to 3 to 4 days interval. The highly mentioned instant messaging platforms were WeChat and Weibo, whereas pregnancy apps were *BabyTree* and *MeetYou*. With regard to the seeking behavior, 18 codes were merged into 4 themes, namely gravida, fetus, delivery, and postpartum period. We identified 7 subcategories within the gravida category, 4 subcategories within the fetus category, 4 subcategories within the delivery category, and 3 subcategories within the postpartum period category. Table 2 presents these details. Among them, the most frequently sought information was related to fetal development, with 90% (18/20) of participants mentioning this while using social media during pregnancy. Nutrition during the postpartum period was the least mentioned, with 10% (2/20) of participants recalling their search. In addition, pregnancy taboo, nutrition during pregnancy, sex of fetus, and pregnancy-related complications were highly cited.

**Table 2:** Pregnancy-related information seeking categories, subcategories, and excerpted examples.

Categories		Number of mentions by participants	Example quotes
**Gravida**			
	Pregnancy taboo	15	“I learned that I can eat shrimp but not crab via the *Health Mothers*.” (WeChat public account)
	Nutrition during pregnancy	12	“I asked in the WeChat group what vitamins they were supplied.”
	Pregnancy-related complications	10	“I was too frightened of encountering gestational diabetes, and I could not help but search it everywhere…”
	Antenatal care	8	“The schedule of pregnancy check on *MeetYou* was what I wanted exactly.”
	Pregnancy symptoms	6	“Although the doctor said that intense vomiting is not a problem, I still searched comments from others with similar conditions for comfort.”
	Amusement and sports	6	“Sometimes I just felt bored and browsed other gravida’s posts to see if they had anything interesting happening.”
	Super-cool pregnancy-related goods	3	“I found a comfort pillow on the *Little Red Book*. It was worth it!”
**Fetus**			
	Fetal development	18	“…It informed me when my baby began to have a heartbeat and could hear me…I followed the app since my pregnancy.”
	Sex of fetus	12	“The doctor did not tell me, I searched clues from other mothers’ experiences.”
	Antenatal training	8	“I played the music in *Baby Tree.*”
	Baby’s name	7	“I looked into answers about their naming techniques.”
**Delivery**			
	Preparation for delivery	12	“I started to compare the maternity packages on the platform since my seven-month pregnancy.”
	Mode and process of delivery	8	“When I learnt of the term painless labor, I searched it for further details.”
	Delivery stories	4	“I am scared about delivery, and luckily found a lot who were sharing their experiences on *MeetYou*.”
	Hospital and doctor choices	4	“I looked at many comments online and choose the doctor I favored.”
**Postpartum period**			
	Infant care	8	“I also compared the mother-baby care center on social media…”
	Maternal recovery and sex life	5	“I looked particularly for information on weight loss and muscle exercises.”
	Nutrition during postpartum period	2	“I hardly checked the nutrition and vitamins after childbirth.”

With regard to the sharing behavior of expectant mothers, public sharing of pregnancy-related information was hardly seen on social media. However, some participants expressed their habit of sharing within closed groups and engaging in private conversations with friends or relatives on social media channels. A total of 10 codes were identified, which emerged into 4 themes, namely, gravida, fetus, delivery, and caretaker. Furthermore, 4 subcategories within the gravida category, 2 subcategories in fetus, delivery, and caretaker, respectively were found. In Table 3, further details are provided. Among them, the most shared information related to their husbands (10 out of 20 participants), whereas the sex of fetus (2 out of 20 participants) was the least referred to topic. In addition, announcement of pregnancies, preparation for delivery, pregnancy symptoms, mode and process of delivery, and other topics about their mother-in-law/mother were also highly discussed on social media.

**Table 3:** Pregnancy-related sharing categories and subcategories.

Categories		Number of mentions by participants
**Gravida**		
	Pregnancy announcement	9
	Pregnancy symptoms	6
	Pregnancy-related complications	5
	Super-cool pregnancy-related goods	5
**Fetus**		
	Fetus pictures	3
	Sex of fetus	2
**Delivery**		
	Preparation for delivery	8
	Mode and process of delivery	6
**Caretaker**		
	Husband	10
	Mother-in-law/mother	6

### Preferred Seeking and Sharing Patterns

#### Lurking on Social Media

On the basis of our interviews, 90% (18/20) of participants noted that they review pregnancy-related information on social media. As one participant stated:

I had a strong prenatal reaction with continued vomiting and dizziness, much stronger than my mother-in-law and my mother had experienced or expected...I wasn’t assured until I found that many similar cases were uploaded by other expectant mothers…I saw one recommendation of several public accounts on social media for pregnancy-related information, and I subscribed to some of them for regular updates.

In addition, 95% (19/20) of participants had not actively engaged in pregnancy-related conversations initiated by other strangers on social media. As one participant expressed:

I do not want to talk with other pregnant women I am not familiar with. I think pregnancy is a personal matter and I do not want to share too much with others I do not know. If I cannot find the health information I need, I will quickly switch to other channels or online conversations to look for more precise answers.

#### Small Group Sharing

All participants believed that they did not need to publicly share information about their pregnancy on social media app. They perceived pregnancy to be a private affair, feeling reluctant to share their news with everyone. However, many participants said that they would use social media channels to connect with close female friends, particularly those who are pregnant, and they showed varying degrees of dependence on pregnancy groups hosted on social media channels. As one participant mentioned:

I was not a social media fan, nor did I want others know about my pregnancy. I felt it unnecessary to post it on social media, and I have not seen any of my friends ever post their pregnancy news on social media…But, I like to share it privately with my girls via WeChat. We have a WeChat group with some pregnant women. We talk about everything during our pregnancy, every day. Sometimes, we Tucao (uncover some foolish story) about our husbands. I felt much relief chatting with them.

#### Bad News Avoidance

Notably, although participants acknowledged the importance of obtaining health information via pregnancy apps, half (10/20, 50%) of the participants also tried to avoid bad news posted by others. For example, one participant revealed that:

This is my first pregnancy and I am very worried about my medical reports, as my doctor said that two core factors are relatively lower, considering my situation. The doctor instructed me to take pills every day during pregnancy, as my diagnosis was preabortion. I was so scared regardless of my doctor’s explanation. Then, I went on to the Meetyou application and joined a chat group that featured my core indictors. Some of the conversations really horrified me. Meanwhile, when I compared my situation with theirs, I felt lucky. One said that this can never be a small thing, and her last child abortion was a lesson.

This feeling was confirmed by another participant who stated that extreme cases were frequently discussed on social media channels; however, most pregnant women do not possess serious conditions. She also added that usually women over the age of 35 years had more serious concerns, as well as those who had previously experienced child abortion.

#### Cross-Checking of References

All participants applied similar search strategies, often searching for the same pregnancy-related information multiple times to obtain a comprehensive understanding of the problem encountered. For example, if an answer received more likes or follows, it could be deemed more convincible. In addition, if the online description of the problem coincided with their real experiences, many participants tended to believe them. If many sources presented similar results, they were likely to be right. One participant stated:

When I searched my symptoms online, I compared the results on different sources. With social media, I can easily see the number of followers, comments, as well as the identity of the providers. For example, I visited Zhihu, a knowledge exchange social networking website, to find out the reason why I became very itchy after several months of my pregnancy. I looked at one conversation on this topic and saw a lot of answers. I can easily identify the reliable ones by comparing the number of Zan (followers). On Zhihu, some people claimed that they were experienced doctors of Obstetrics and Gynecology. I valued their information very much.

### Impact of Social Media Use

In general, participants were satisfied with pregnancy-related information found via social media channels and reported a positive impact. All participants agreed that the health information they had found via online channels had greatly empowered them to make more informed decisions during pregnancy. Overall, 95% (19/20) of participants were moderately or very satisfied with the current pregnancy-related provision. For firsthand knowledge on pregnancy-related topics offered via social media, participants were much appreciative:

My mother used to tell me about her experience. However, I found that was not practical for me…Thanks to social media, I can connect with my friends who have experienced childbirth. Also, with the range in applications, I can know almost everything going on in my body during the whole pregnancy period.

In addition, 95% (19/20) of participants reported a positive impact from using social media during their pregnancy. Only 1 participant stated that no impact had occurred during pregnancy; however, all primipara participants expressed that health information found via social media was of great value for them and made their first pregnancy a smooth experience. Furthermore, they also believed that social media had created a communicative platform for allowing them to chat with other pregnant women and get timely support. This contributes to the reduction of anxiety and loneliness during pregnancy. As one participant said:

Social media not only helps me with finding pregnancy-related information, but it also makes me more confident about my pregnancy and puts me in a good mood…If I get bored, I can read funny stories or play small virtual games embedded on the MeetYou application, to kill time.

## Discussion

### Principal Findings

This study aimed to understand the social media usage of pregnant women in China, including its adoption, manifestation, and its impact. Similar to studies conducted in developed countries [12,23], this study, focused on China, the largest developing country, has also employed a qualitative approach to reveal the status quo of social media use for pregnancy-related purposes.

First, the frequency of using social media and the topics concerned are distinct between seeking and sharing behaviors among Chinese expectant mothers. This study revealed that seeking happened more frequently than sharing on social media. This may attribute to the ease of the 2 behaviors. For seeking behavior, it does not involve new knowledge or information creation. In the case of seeking pregnancy-related information, the process of information retrieval could be achieved in minutes. However, in the case of sharing, it creates new information, takes time before sharing, and has almost immediate consequences. Sharing pregnancy-related information requires efforts in organizing language as well as concerns for safety and privacy. The high utilization of social media for pregnancy-related information seeking means that pregnant women in general have insufficient knowledge about their pregnancy and have a general anxiety; this is especially the case for first-time expectant mothers. The increasing division between provision and demand of pregnancy-related information may contribute to the frequent use of social media. From the perspective of provision, this implies that there is a failure in health education for pregnant women during the new baby–born peak, when China abandoned the 1 child policy. It is worthwhile for hospitals or other medical institutions to utilize various means to engage with expectant mothers who are seeking health-related knowledge, for example, providing the latest pregnancy-related information on popular social media channels, including Weibo, WeChat, and short-video platforms, to reach a large audience of expectant mothers.

With regard to the topics, seeking behavior focused on the expectant mother and fetus, whereas the postpartum period was less concerning, which coincides mostly with previous studies on Chinese pregnant women seeking health-related information [5]. In addition, several new seeking intentions appeared, such as amusement, sex of fetus, and super-cool pregnancy-related goods. This variation may imply a change in perception of pregnancy, calling for more enjoyable pregnancy experiences rather than merely safe pregnancy. As for information sharing, the most mentioned topics were related to husband and pregnancy announcement. This is different from the sharing habits of pregnant women in the United States, where information about husbands is not on their sharing list [23]. With regard to sharing information specifically about their husbands, the most highly mentioned topic related to foolery or funny things happening during the caretaking process by the husband. This sharing could be interpreted as fun or anxiety relieving to gain peace of mind [24].

Second, pregnant women are silent on public social media channels in general but do actively engage in small online groups. Traditionally, pregnancy in Chinese culture is a personal affair and one which is shared only in closed groups. This may attribute to the general notion that pregnancy is a vulnerable state in Chinese culture [25]. In particular, pregnancy is regarded as high-risk within the first 3 months; thus, Chinese pregnant women often do not share their news openly on social media during this early stage, rather they prefer to announce their pregnancy only to close relatives and friends after their condition has become stable. Usually, the husband is the first to learn of the pregnancy, then other family members or close friends are informed. A comparative study between Australian and Chinese pregnant women showed a significant cultural difference in terms of beliefs, attitudes, barriers, and intentions toward exercise during pregnancy [26]. When considering pregnancy taboos that are deeply rooted in Chinese culture, expectant mothers in China are generally much more careful than those in Western countries [14], which leads to their lack of engagement on social media.

Previous anthropology studies have demonstrated that the sharing nature of women has been passed on through generations and historical experiences, such as childbirth, child rearing, and feeding the family and guests [27,28]. For pregnant women, their ability to share or engage in conversation is evident on social media, but in a much more private manner. This may be because of the hierarchy of trust as indicated by Fei et al [29], where closer relationships deserve more trust. Compared with random conversations with pregnant women they are not familiar with, Chinese expectant mothers prefer to share their concerns, talk about their experiences, and offer instant help within their social circle of close family members or friends who share childbearing experiences. In addition, certain pregnancy-related inquiries could be deemed intimate, such as questions relating to sexual activity during pregnancy. A possible strategy for social media–based prenatal care would be to encourage participation for sharing information by initiating public conversation and inviting opinions from leaders, for example, health professionals in renowned hospitals or people with influences in the online community. Increased participation in return brings more users and, thus, increases the market.

Third, avoiding bad news and cross-checking reference were involuntary seeking habits among Chinese pregnant women. Pregnant women have a tendency to avoid bad news presented by others online and refuse to read the details on subjects such as miscarriage or stillbirth. Two explanations for this phenomenon are fear and sensitivity. The physical changes experienced by women during pregnancy lead them to a heightened awareness of their surrounding environment. If a negative story is found, pregnant women could easily relate this to their own situation and put themselves in the mind of the storyteller; for example, a topic on nausea and vomiting during pregnancy posted on social media. The online comments could be varied relating to the etiology and corresponding treatment [30], which may involve extreme cases and bad outcomes. A review of them is likely to cause discomfort and fear. This is consistent with previous studies that have explored negative stories surrounding childbirth, pregnancy, or baby care, which could result in stress, effects on everyday life, and avoidance of childbirth or pregnancy altogether [31]. Despite the internal feelings experienced when reading such stories, negative outcomes could also be related to interpersonal or service-related problems such as tension in the patient-clinician relationship or postponing a clinical encounter [32]. Risk aversion is in the nature of human beings and this also applies to pregnant women. However, there is no guarantee to keep pregnant women from experiencing any negative outcomes as the information found on social media is hard to avoid. Health professionals or supporters of social media usage during pregnancy could help expectant mothers establish scientific mindsets to reduce the negative outcomes from social media usage; for example, expectant mothers could share and discuss with their physicians.

Our study also showed that the evaluation of health information by pregnant women followed similar principles: searching, but never fully sourcing it from one place. This may be because of the chaotic nature of pregnancy-related information provided online, where no specific standards are currently implemented. As not all pregnancy-related information on social media is deemed helpful, pregnant women must be cautious in applying the knowledge they retrieve in their daily lives. With constant criticism from media about false or fake information from social media channels, pregnant women in China are quite aware of the side effects of social media. As pregnancy in Chinese culture is a critical family business rather than a personal experience, pregnancy attracts great attention either from the expectant mother or from her family [33]. There is no doubt that a small problem experienced during pregnancy could be amplified, requiring careful checking for consistency in information obtained from social media using an offline approach such as seeking health professionals’ advice. Indeed, through decades of efforts in national prenatal care, the general health literacy level among pregnant women has increased significantly [34], which also makes pregnant women more confident in using online pregnancy-related information.

Finally, although reported impacts were positive, the digital divide of providing pregnancy-related information deserves practical attention. Although almost all participants in this study claimed a positive result in using social media during pregnancy, it cannot be assumed as a generalized conclusion that social media provides benefits for all pregnant women on all occasions in China. Many pregnant women with a lower level of education, poor health literacy, and unemployment do not use social media for pregnancy-related purposes. As this group of pregnant women align to the profile of expectant mothers living in rural areas of China, with access to limited medical resources and a neighborhood with all known acquaintances, they do not feel the need or desire to use social media for pregnancy-related purposes as often their relatives and friends, who have experienced childbirth, live close by and can share their experiences on a personal level. Some, in fact, may go to a hospital for the purpose of delivery only. In our study, the participants were mostly highly educated, comparatively affluent, representing the largest group of users among all pregnant women in China. The positive outcomes experienced from social media is likely to be connected with their comparable advantages in internet expertise and health information literacy [35]; this may lead to a *digital divide* in the quality of health care provision among pregnant women with different socioeconomic statuses. To maximize the benefit of social media for expectant mothers, it is worth employing marketing strategies that target pregnant women with lower literacy or lower incomes and exploring further social media–enabled online consultation methods designed specifically for these users [11].

### Conclusions

This study investigated the current adoption and behavioral habits of using social media by Chinese expectant mothers when seeking and sharing pregnancy-related information. We interviewed 20 pregnant women, applying thematic analysis to summarize the major themes regarding how pregnant women use social media and the perceived impact of its use. Generally, pregnancy-related information seeking behaviors are more frequent than sharing behaviors. The top pregnancy-related information seeking and sharing topics were related to fetal development and about the expectant father during pregnancy. In terms of specific patterns, lurking, small group sharing, bad news avoidance, and cross-checking of references were identified. Most expectant mothers reported positive outcomes from social media usage for pregnancy-related purposes.

Our study had some limitations. First, all participants were healthy without any complications accompanied with their pregnancy [36], such as being overweight or having hypertension or type 2 diabetes. For pregnant women with complications, their condition may be major, making them more likely to be unsatisfied about the medical consultation received, which may lead to a different pattern of social media usage and impact. A focus on pregnant women with specific conditions and various severities may desire future exploration [37]. Second, this study concerns pregnant women in China exclusively and did not distinguish between women whose medical resources are constrained and those with abundant medical resources. Further research could explore those with medical resource deficiency and compare pregnant women in such areas with those having affluent resources. Finally, this research may have overlooked the other online seeking habits and sharing behaviors of pregnant women, which could be of critical importance; for example, their sentiments with their husbands, mothers-in-law, and other caretakers [1,15].

There is no doubt that by using social media, Chinese expectant mothers are extensively empowered to realize and seek better prenatal care. Thus, social media not only serves to provide health knowledge for pregnancy safety but also guides pregnant women during their journey to motherhood, being wary of their own situation and becoming more joyful during their pregnancy [24].

## Abbreviations

RMB: Renminbi
